# When ‘Sanctity of Life’ and ‘Self-Determination’ clash: Briggs versus Briggs [2016] EWCOP 53 – implications for policy and practice

**DOI:** 10.1136/medethics-2016-104118

**Published:** 2017-07-22

**Authors:** Jenny Kitzinger, Celia Kitzinger, Jakki Cowley

**Affiliations:** 1 School of Journalism, Media and Cultural Studies, Cardiff University, Cardiff, UK; 2 Department of Sociology, University of York, York, UK; 3 Empowerment Matters, Liscard Business Centre, The Old School, Liscard, UK

**Keywords:** Capacity, Prolongation of Life and Euthanasia, Quality/Value of Life/Personhood, Autonomy, Decision-making

## Abstract

In a landmark judgment in the English Court of Protection, the judge (Charles J) found it to be in the best interests of a minimally conscious patient for clinically assisted nutrition and hydration (CANH) to be withdrawn, with the inevitable consequence that the patient would die. In making this judgment, it was accepted that the patient’s level of consciousness — if CANH were continued and rehabilitation provided — might improve, and that he might become capable of expressing emotions and making simple choices. The decision to withdraw treatment relied on a best interests decision, which gave great weight to the patient’s past wishes, feelings, values and beliefs, and brought a ‘holistic’ approach to understanding what this particular patient would have wanted. We draw on our own experience of supporting families, advocating for patients and training healthcare professionals in similar situations to consider the implications of the published judgment for policy and practice with patients in prolonged disorders of consciousness and their families.

In 2016, a landmark and widely reported judgment in the English Court of Protection^1^ found it to be in the best interests of a minimally conscious* patient for clinically assisted nutrition and hydration (CANH) to be withdrawn, with the inevitable consequence that the patient would die. In this commentary, we consider the implications of the judgment for policy and practice with patients in prolonged disorders of consciousness† and their families. We are particularly concerned to address how the judgment can inform the legal consciousness and moral reasoning of people who care for and about these patients in everyday practice.

The facts as recorded in the published judgment^1^ are as follows: Paul Briggs (PB), a police officer, was knocked off his motorbike while travelling to work in July 2015. He was diagnosed as being in a minimally conscious state (MCS) at the point at which the case was heard and judgment handed down in December 2016. This was more than 16 months after his traumatic brain injury, but well within the 5-year post-onset period during which it is clinically accepted that improvements in the level of consciousness can occur.^2^ Proceedings were brought by his wife, Lindsey Briggs, who believed that her husband would not want his life to be prolonged in his current — and likely future — condition. She was opposed by the NHS Trust, the Clinical Commissioning Group responsible for providing medical treatment and the Official Solicitor (PB’s ‘litigation friend’), all of whom argued that it was in PB’s best interests to receive further assessment and treatment with the possibility that his degree of consciousness might improve, although with remaining profound physical and mental disabilities such that it was highly unlikely that he would ever be able to make his own serious medical treatment decisions.

## Best interests

Those unfamiliar with the English legal system for adults who lack capacity should note that, in accordance with the Mental Capacity Act 2005, all parties were required to address the question of PB’s ‘best interests’ rather than seeking to apply the standard of ‘substituted judgment’, that is, what the patient might have decided for himself if he’d had capacity to do so (which is the standard applied in many other jurisdictions). ‘Best interests’ include consideration of the person’s past and present wishes and feelings, and their values, beliefs and any other factors they would consider as relevant to their decision if they were able to do so (s 4 (6) Mental Capacity Act 2005), but also puts weight on ‘all the relevant circumstances’ (s 4 (2) Mental Capacity Act 2005), which might — and, in practice, regularly do — include diagnosis, prognosis and ‘sanctity of life’.

The relative weight to be given to a person’s own views in arriving at a ‘best interests’ decision and how to set those against ‘sanctity of life’ is not specified in the Act, but has developed through case law. Public understanding of the application of the law to minimally conscious patients (and the weight to be given to the person’s own views) is informed by an earlier (highly publicised) judgment, *W v M & Ors [2011]* EWHC 2443 (Fam), in which, as in *Briggs,* family members were seeking authorisation for CANH withdrawal from a minimally conscious patient and were opposed by the Primary Care Trust and Official Solicitor. In that case, the presiding judge (Baker J) accepted the family’s reports about the patient’s general views about matters such as life-prolonging treatments and institutional living, but took a narrow approach to interpreting her prior wishes, highlighting the fact that she had not specifically said that if she were in an MCS she would want CANH withdrawn.‡ He also said it would ‘be wrong to attach significant weight to those statements made prior to her collapse’ when setting them against ‘the importance of the sanctity of life’. He ordered that treatment must be continued. In the aftermath of this judgment, there was a widespread belief among family members of minimally conscious patients that CANH withdrawal could not be authorised for patients so diagnosed, and this view was reinforced by some legal advisors and healthcare practitioners.^3 4^ The *Briggs* case is hugely significant, then, for its potential to reverse the effect of *W v M* and for the signal it sends to everyone involved that CANH may not be in the best interests of all MCS patients.§

## The significance of *Briggs* for everyday practice

The *Briggs* case was described by the presiding judge as one that engages fundamental principles: ‘on the one side, the sanctity of life and so the very strong but not absolute presumption in favour of continuing Mr Briggs’ life and, on the other side, the principle of self-determination’ (para 8).^1^ Framing up the issue in this way makes *Briggs* (like *Bland*,^5^ the first UK case to authorise withdrawal from a patient in a permanent vegetative state) a quintessential ‘stigmata’ case. It ‘enables the marks of a deeper system of values to be seen on the surface of a specific dispute’^6^ and ‘provides a context in which the values of a society can be expressed and also a mechanism whereby clashes of competing values can be resolved’.^7^ The *Briggs* judgment raises and engages directly with ethical arguments accessible and relevant to the concerns often expressed by families and healthcare professionals (and indeed in public debate) about similar dilemmas in relation to other patients.

The *Briggs* judgment is significant in part for the great weight Charles J gave to the person’s own views, even when set against ‘sanctity of life’: ‘if the decision that P would have made, and so their wishes on such an intensely personal issue can be ascertained with sufficient certainty it should generally prevail over the very strong presumption in favour of preserving life’ (para 62).^1^ This is in keeping with the ‘readily discernible’ trend other commentators have noted when examining the evolution of case law in the Court of Protection.^8^

The facts in *Briggs*, the priority given to ascertaining his wishes and the decision reached, are particularly significant given that large numbers of patients in disorders of consciousness¶ are maintained in care homes for many years without any consideration as to whether or not continuing CANH is what they would have wanted. Previous research shows this is so even when their families believe that the person would want to refuse treatment and have tried to communicate this fact to staff.^9^

‘Best interests’ decisions — including any effort to ascertain or factor in the patient’s own wishes — are often put ‘on hold’ until a certain level of diagnostic and prognostic certainty has been reached, during which time families are not asked about patients’ views about continuing CANH on the spurious grounds that CANH *must* be delivered until the prognosis is clearer. For some of these patients (those who emerge), there is then no treatment to withdraw (because swallowing has been established and CANH is no longer required) and the’ window of opportunity’ for allowing death by treatment withdrawal has passed.^9^

The key competing concepts of ‘sanctity of life’ and ‘self-determination’ invoked and deployed in the *Briggs* case are deeply resonant for families and healthcare professionals. Conflicting personal commitments and contested claims about the legal and ethical status of these principles are mobilised in deciding the fate of the much larger number of patients whose cases never reach the court — in relation to CANH and also in relation to other life-prolonging treatment decisions concerning, for example, resuscitation in the event of cardiac arrest or the provision of antibiotics for life-threatening infections. This makes the *Briggs* judgment very important for opening up discussion — and indeed invaluable, as a training tool — not only regarding the care of MCS patients, but also in relation to patients lacking capacity in a wide range of other conditions. We use the case to inform reflection in face-to-face training in care homes and are integrating it into an online e-learning resource.

In what follows we highlight the ways in which the *Briggs* judgment can be used to inform and engage families and professionals with respect to the two key principles invoked, and the balance between them.

### ‘Sanctity of Life’

The concept of ‘sanctity of life’ has been extensively analysed by philosophers for whom it can seem ‘impossibly vague and misleading’,^10^ but the judicial use of the phrase in *Briggs* (on each of its 10 occasions of use) seems simply to refer to a strong (but not absolute) presumption in favour of preserving human life. For example: ‘The default position for such persons is founded on the sanctity of life and so the strong presumption that lives that have value should be continued by life-sustaining treatment (here CANH)’ (para 3).^1^

The term ‘sanctity of life’ is often understood as a religious concept, and we have found it rarely used in practice either by families or by health practitioners except those speaking as members of a faith group. Nonetheless, people very commonly display a commitment to a secular version of ‘sanctity of life’ (like that of the *Briggs* judgment) when they express the view that it would be wrong to ‘cause’ death by withdrawing treatment. It can feel to both families and healthcare staff as though CANH withdrawal (in particular) is a form of ‘euthanasia’ and it is sometimes associated with ‘assisted dying’.^11^ Some believe it to be unethical or unlawful (even when they accept withdrawal or withholding of other life-prolonging treatments) because nutrition and hydration are seen as basic care. Some believe it violates professional ethics, the Hippocratic Oath, or the ‘duty of care’. The *Briggs* judgment is helpful in opening up debate around these issues because Charles J is clear that ‘this is not an assisted dying or euthanasia case’ (para 19).^1^ Nonetheless, for some professionals and families ‘sanctity of life’ considerations (and the enduring hope for future recovery, however unlikely or however limited) will over-ride all other considerations — and, indeed, some patients may have held this position themselves when they had capacity to do so.

On the other hand, it is not unusual for family members to express the view that their relative ‘would rather be dead’, that they ‘wish he had died’ or that they ‘hope she will be at peace’ soon.^9^ Health professionals tend to react with alarm to such statements, not wanting to be implicated in ‘causing’ death and sometimes aware that, in law, a ‘desire to bring about [the patient’s] death’ is specifically prohibited as a ‘motivation’ on the part of the decision-makers in considering whether treatment is in the best interests of the patient (s 4 (5) Mental Capacity Act). Charles J pointed out that Lindsey Briggs’ case was based ‘on what Mr Briggs would have wanted, and that wish has been expressed in terms that he would have wanted to die’ (para 89).^1^ The very fact that it was expressed in this way was used by the counsel for the Clinical Commissioning Group and NHS Trust to argue that this precluded the court from making a decision in favour of treatment withdrawal. This was not an argument that the judge accepted. Instead he pointed out that the court (post-*Aintree*) was required to decide whether or not continued treatment was in PB’s best interests, and a decision that it was not would *not* be motivated by a desire to bring about PB’s death, notwithstanding the fact that PB — if he had had capacity — might have been so motivated, as his wife claimed he would have been (para 94).^1^ One clear implication of the *Briggs* judgment, then, is that health professionals need not censure or recoil from family members who express the view that their relative ‘would rather be dead’. Rather this should be treated as an opportunity to involve families in best interests decision-making and to explore what the patient’s treatment wishes might have been.

### Self-determination

The term ‘self-determination’ (used on 15 occasions in the judgment) refers simply to ‘what [Mr Briggs] would have wanted to do’ (para 7).^1^ Stated in more general terms, the term is used to mandate an enquiry into ‘what decision he or she would have made if they now had capacity and so, in exercise of their right of self-determination was able to make the decision’ (para 69).^1^ ‘Self-determination’ is only one element of ‘best interests’ decision-making, but — following *Briggs* — a very important one: ‘having his views and wishes taken into account and respected is a very significant aspect of P’s best interests’ (para 56).^1^

The judge’s focus was clearly on PB’s views and wishes *as he would have expressed them when he had capacity* and not on his current wishes regarding treatment (since none were ascertainable), nor on wishes that he might possibly develop in the future if continued treatment and rehabilitation were to lead to a higher level of consciousness (although still lacking capacity to actually make the decision himself). There is a substantial philosophical literature addressing the question of what weight to give to past (capacitous) wishes if current (non-capacitous) wishes conflict with them,^12 13^ but this literature is not immediately relevant here since PB had no current discernible wishes or feelings (although it is possible that he may have developed some if treatment had been continued). Nevertheless it is worth noting that concerns about hypothetical future wishes are often integral to family and staff thinking on the ground in similar cases. Families sometimes hope that the person might recover sufficiently to express their own views, and hence relieve them of responsibility for contributing to treatment decisions. Families also report having been told that they should be open to accepting a ‘new’ post brain injury person in place of the ‘old’ person they knew before — and that profound brain injury may ‘change the mind’ of the brain-injured person such that their past wishes have limited relevance, and the person might even be happy in a situation they had previously said would be intolerable to them (as it was claimed was likely to be the case for PB). Individuals take different perspectives on this issue,^14^ and families can be asked to consider what weight *the patient* (when capacitous) would have place on the wishes and feelings of a possible future non-capacitous self.

Since ‘self-determination’ for vegetative and minimally conscious patients depends crucially on ascertaining what patients’ views would have been when they had capacity, it is essential to elicit these in a timely fashion from those ‘engaged in caring for the person or interested in his welfare’ (s 4 (7)(b) Mental Capacity Act) — most likely the person’s family but also potentially (as in *Briggs*) the person’s friends and colleagues. Most families report that they have never been asked whether or not the patient would want continuing CANH treatment: the feeding tube is treated as a ‘given’, and reinserted and replaced without discussion, even when decisions have been taken to limit other potentially life-prolonging treatments.^15^ It takes tremendous courage for families to raise CANH withdrawal with staff.^9^ Those who do find ways of broaching the subject often feel that staff are sceptical about their ability to represent their relative’s wishes or suspect self-interested motives. It is true that the patient’s wishes are rarely immediately apparent, since few people provide specific instructions (equivalent to an advance decision) about what they would and would not want if they were in a disorder of consciousness, so relatives must extrapolate from things said and done in relation to similar but not identical circumstances (eg, in *Briggs*, PB’s support for his mother-in-law’s refusal of a feeding tube when she was dying with cancer). But the ‘holistic’ approach displayed in the *Briggs* judgment took as evidence not just what he said, but how he lived his life and embodied his values through the choices he made of, for example, employment, leisure pursuits and other displays of who and what mattered to him. Charles J points out that PB was ‘a committed and loving family man’ and that what he might want can reasonably be assumed to include ‘the interests of other people who P would have been likely to take into account’, that is, the interests of his wife and child (para 56).^1^ Health staff should feel reassured that when family members asserting that their relative would rather be dead say things like ‘he wouldn’t want to put *us* through this’, the reported concern of the patient for the well-being of his family can legitimately be part of the best interests decision-making process.

## Conclusion

### Best interests decision-making after *Briggs*

According to Charles J, the decision that patients would have made regarding life-prolonging treatment when they had capacity should prevail over the presumption in favour of preserving their lives. This places a very strong requirement on those delivering treatments to determine, as best they can, what the patient’s decision would have been. As should be obvious, the absence of a formal written advance decision cannot be used to claim that the patient has no ascertainable views, or (worse) that since the patient has not formally refused treatment consent can be presumed. Our broader research shows little evidence of much enquiry into patients' wishes for the vast majority of those in prolonged disorders of consciousness. Following *Briggs* such enquiry would require the following:

1. *Developing a ‘holistic’ understanding of who the patient was before he lost capacity* by listening to family and friends/colleagues with the same compassion and attentiveness that Charles J displayed in *Briggs* (or employing an independent patient advocate to do so). The judge evaluated the evidence the family had presented by asking ‘whether the man described to me, and so a man with his beliefs and values and approach to life would consent to his CANH treatment if he had heard the evidence and argument before me’ and detailed his understanding of PB as a Gulf War veteran and police officer, ‘a risk taker and a man of courage’ (para 120), and a committed family man. He engaged with the best interests decision as it relates to *this particular person in this particular situation* and considered what *PB in particular* would decide for himself if he were able so to do.
2. *Giving the family as clear a sense as possible of the patient’s current condition and realistic ‘best case scenario’ in the future, if CANH is continued and any further rehabilitation provided. *Families are rarely provided with this information at present: they have sometimes simply been told that ‘anything is possible’ or ‘we just don’t know’, and some are still hoping, years later, for ‘miracle recoveries’ of the type regularly featured in the media. The person’s likely future needs to be explained in ordinary language and in specific detail (ie, not simply ‘very disabled’). In *Briggs,* the realistic ‘best case scenario’ was established via a report agreed between the treating clinician and the expert rehabilitation consultant: PB would not regain mental capacity to make complex decisions (eg, about his own medical treatment, or even about whether to wear a jumper or a t-shirt based on an assessment of the weather), but he could be happy, make simple choices (eg, choose between a blue t-shirt and a red t-shirt) and would have some pleasurable — and some painful — experiences: he would be severely physically impaired needing 24-hour care and dependent on others for all activities of daily living (para 51).^1^ Recovery beyond this state was ‘not impossible only in the sense that one should never say never’ (para 47).^1^
3. *Attending to family and friends’/colleagues’ views as to what the patient would have wanted, based on an understanding of his current state and ‘best case scenario’ future (and taking into account attitudes to risk and uncertainty).* Elicitation of these views should avoid ‘pedantic’ and ‘unsympathetic’ cross-examination (para 97)^1^ and acknowledge that different family/friends may see things from slightly different perspectives. Despite differences between the way family members expressed themselves and the varying times at which they had arrived at their conclusion, the Briggs family was ‘convinced that if he was able to express it his view would be ‘enough is enough’ because … for him this [i.e. even the best case scenario] was not a life that was worth living’ (para 112).^1^ Family members also pointed out that ‘Mr Briggs would factor in the point that the hoped for improvement to the best case scenario is only a possibility’ (para 116).^1^

In conclusion, the *Briggs* case is significant because of its role in case law as a precedent for future decision-making by the courts and because the arguments used in court address core ethical issues in everyday thinking on the part of those who care for and about these patients. This makes it very different from some judgments concerning these patients that have focused more narrowly on the patients’ precise diagnosis as a prerequisite for decision-making — often not of primary concern to the family, who believe that the patient would not want to be kept alive in either a vegetative or a minimally conscious state.^14^ The *Briggs* judgment, and the reasoning behind it, should change the texture and quality of best interests decision-making about these patients ‘on the ground’. It does this by showing in the clearest possible way that the patient’s prior wishes, feelings, values and beliefs carry a great deal of weight in best interests decision-making, and by offering a model for how such patient-centred decision-making can be implemented. Neither sanctity of life nor uncertainty of diagnosis/prognosis is an automatic trump card dictating life-prolonging treatment. Rather, the individual person and what they would have decided when they had capacity must be at the centre of all decisions made about them.

## Footnotes

* The ‘minimally conscious state’ (MCS) is defined as ‘a state of severely altered consciousness in which minimal but clearly discernible behavioural evidence of self- or environmental awareness is demonstrated. MCS is characterised by *inconsistent, but reproducible*, responses above the level of spontaneous or reflexive behaviour, which indicate that the patient has some degree of interaction with their surroundings’.^2^

† Prolonged disorders of consciousness include ‘coma’, the ‘vegetative state’ and the ‘minimally conscious state’ (but not ‘locked-in syndrome’ or ‘brainstem death’). Full definitions can be found in the National Clinical Guidelines.^2^

‡ The *W v M* case preceded the judgment in *Aintree University Hospitals NHS Trust v James* [2014] AC 509, in which the Supreme Court confirmed that a much more holistic approach (as now exemplified in *Briggs*) is to be taken to understanding patients' wishes.

§ There are two earlier cases in which judges authorised withholding/withdrawing of CANH from patients diagnosed as being in MCS, but the facts in these cases are significantly different in ways that favoured a decision *against* continuing treatment. In one the patient had been physically resistant to all efforts to provide her with nutrition and had pulled out a nasogastric tube and several cannula (*United Lincolnshire Hospitals NHS Trust v N* [2014] COPLR 60). In the other the patient had very advanced multiple sclerosis (*Re N* [2016] COPLR 88). In *Briggs*, by contrast, there were no practical impediments to continued CANH, and he did not have a terminal condition.

¶ There is no central register of such patients, so accurate reports of their numbers are impossible to obtain — and doubtless many are undiagnosed or misdiagnosed — but estimates suggest that there may be between 4000 and 16 000 permanent vegetative state (PVS) patients in the UK and up to three times that many in MCS.^16^
